# Acute Care Visits for Assault and Maltreatment Before vs During the COVID-19 Pandemic in Ontario, Canada

**DOI:** 10.1001/jamahealthforum.2021.1983

**Published:** 2021-08-06

**Authors:** Natasha Saunders, Lesley Plumptre, Christina Diong, Sima Gandhi, Michael Schull, Astrid Guttmann, J. Michael Paterson

**Affiliations:** 1 ICES, Toronto, Ontario, Canada; 2 Division of Pediatric Medicine, The Hospital for Sick Children, Toronto, Ontario, Canada; 3 Department of Pediatrics, University of Toronto, Toronto, Ontario, Canada; 4 Child Health Evaluative Sciences, SickKids Research Institute, Toronto, Ontario, Canada; 5 Institute of Health Policy, Management and Evaluation, University of Toronto, Toronto, Ontario, Canada; 6 Edwin S. H. Leong Centre for Healthy Children, University of Toronto, Toronto, Ontario, Canada; 7 Department of Medicine, University of Toronto, Toronto, Ontario, Canada; 8 Evaluative Clinical Sciences, Sunnybrook Research Institute, Toronto, Ontario, Canada; 9 Department of Family Medicine, McMaster University, Hamilton, Ontario, Canada

## Abstract

This cross-sectional study compares rates of emergency department visits and hospitalizations for assault and maltreatment by age category and sex in Ontario, Canada, before vs during the COVID-10 pandemic.

## Introduction

COVID-19–related social isolation, family stress, economic loss, and social service reductions have led to concerns regarding increased risks for interpersonal violence and child maltreatment.^1^ Disruptions to conventional safety nets and supports to prevent individuals from experiencing violence or to facilitate early identification of violence have occurred, including closures of schools, childcare facilities, and community programs.^2^ In response, World Health Organization member states, including Canada, have implemented measures that have been largely government sponsored to prevent or respond to potential increases in interpersonal violence.^3^ Although the threat of experiencing violence remains, the extent to which pandemic measures have been associated with changes in visits to hospitals for violent injuries is unknown. We sought to compare rates of emergency department (ED) visits and hospitalizations for assault and maltreatment in Ontario, Canada, before vs during the COVID-19 pandemic.

## Methods

We conducted a population-based, repeated cross-sectional study of acute care visits among all residents of Ontario from January 1, 2017, to December 31, 2020, in Ontario, Canada. Hospitalizations and ED visits for assault and maltreatment were identified using *International Statistical Classification of Diseases and Related Health Problems, Tenth Revision, Canada* diagnosis codes found in hospital (Canadian Institute for Health Information Discharge Abstract Database) and ED (National Ambulatory Care Reporting System) discharge records (eTable in the Supplement). The Ontario Registered Persons Database, the registry of individuals eligible for Ontario’s universal health insurance program, was used for the population denominator. Data use was authorized under §45 of Ontario’s Personal Health Information Protection Act, which does not require research ethics board review or informed consent. This study followed the Strengthening the Reporting of Observational Studies in Epidemiology (STROBE) reporting guideline.

We computed the crude monthly rate of ED or hospital discharges with a recorded assault or maltreatment diagnosis per 100 000 individuals. We calculated the percentage change in rates during the months after the onset of the COVID-19 pandemic (March 14, 2020) and the mean rates during the corresponding months in the previous 3 years (2017-2019). Children (age, ≤12 years) and adolescents (age, 13-17 years) were assessed separately from adults (age, ≥18 years), and adults were further stratified by sex. For context, we used the same methods and reported all-cause ED visits and hospitalizations. Statistical analyses were conducted using SAS, version 9.4 (SAS Institute Inc).

## Results

**Figure 1.**
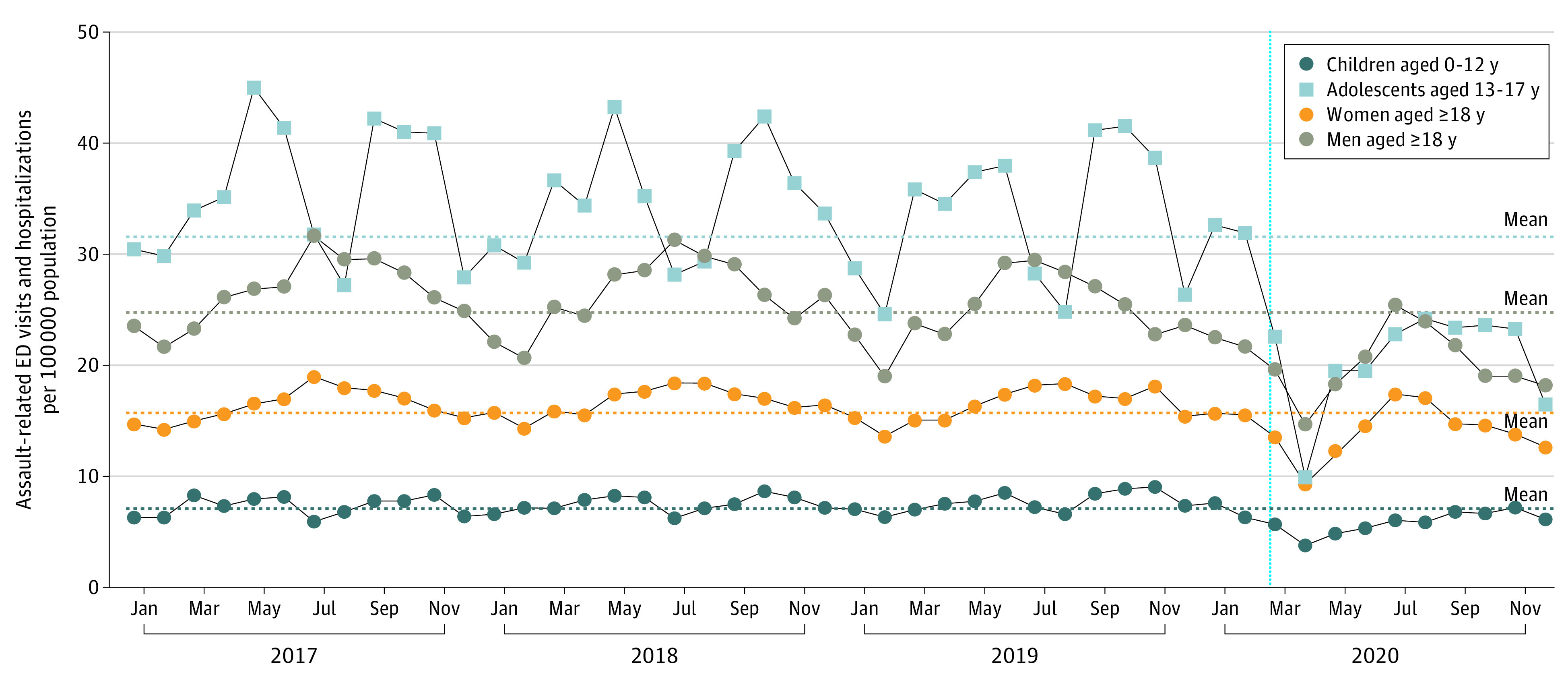
Monthly Rates of Assault-Related Emergency Department (ED) Visits and Hospitalizations in Ontario, Canada, From January 2017 to December 2020 The vertical dashed blue line indicates the approximate onset of the COVID-19 pandemic in Ontario, Canada.

**Figure 2.**
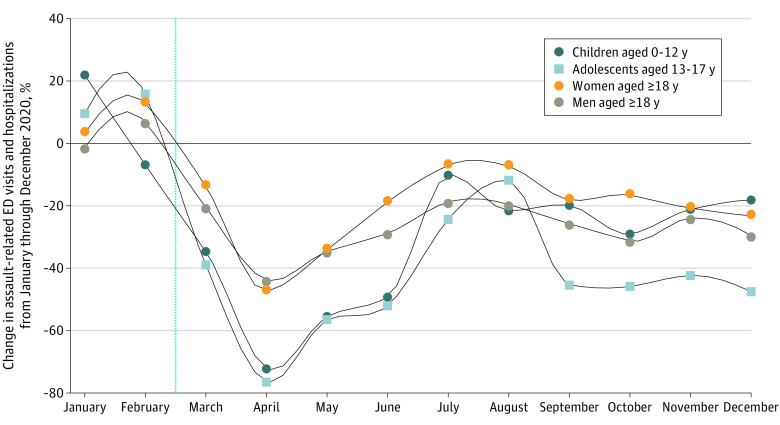
Change in Assault-Related Emergency Department (ED) Visits and Hospitalizations From January Through December 2020 The vertical dashed blue line indicates the approximate onset of the COVID-19 pandemic in Ontario, Canada.

Among 15 067 955 individuals included in the analysis (2 936 459 children and adolescents [19.5%]; 12 131 496 adults [80.5%]), 5134 children (4.2%), 11 796 adolescents (9.7%), 40 437 women (33.2%), and 64 578 men (53.0%) had health record discharge diagnoses for assault or maltreatment after visiting acute care during the study period. The greatest overall volume was observed in July and August among adults, May and June among children, and October and November among adolescents; the mean monthly visit rates were 30.7 per 100 000 population among adolescents, 23.5 per 100 000 population among men, 14.1 per 100 000 population among women, and 5.0 per 100 000 population among children (Figure 1). Compared with the prepandemic 3-year mean for the corresponding month, the rate of visits had decreased by 72% among children, 76% among adolescents, 47% among women, and 43% among men in April 2020 (Figure 1 and Figure 2). In all groups, visit rates were below baseline in July 2020 and again decreased to 18% to 47% of expected visit rates through December 2020. We observed similar patterns for all-cause ED visits and hospitalizations.

## Discussion

In this population-based cross-sectional study of 15 067 955 people in Ontario, Canada, after the onset of the COVID-19 pandemic, the rates of acute care visits for assault and maltreatment decreased immediately and markedly. As of December 2020, rates remained below prepandemic levels in all groups studied. The main limitations of this study are exclusion of injuries with milder physical consequences not requiring hospital care and possible differences in care-seeking behavior during the pandemic, including fewer referrals owing to reduced contact with mandated reporters (eg, teachers, clinicians).

These data suggest that despite theoretical concerns, speculation, and other data sources^4,5^ of increased interpersonal violence, ED and hospital visits for assault and maltreatment did not increase in this region during the COVID-19 pandemic. Similar findings for child maltreatment have been observed in US EDs.^6^ The contributions of specific government interventions to our findings merit further study. Ongoing population-level surveillance with multiple sources of data appears to be warranted to ensure that safety planning needs and services are provided and that risk factors among individuals experiencing violence are adequately addressed.

## Notes

Supplement. **eTable.** Diagnosis (ICD-10-CA) Codes Used to Identify Assault and Maltreatment Related ED Visits and Hospitalizations

## References

[1] Yousif N. ‘Disturbing trend’: Ottawa hospital sees rise in number of babies with severe head injuries during second wave of COVID-19. *The Toronto Star*. January 29, 2021. Accessed May 4, 2021. https://www.thestar.com/news/gta/2021/01/29/disturbing-trend-ottawa-hospital-sees-rise-in-number-of-babies-with-severe-head-injuries-during-second-wave-of-covid-19.html

[2] Metheny N, Perri M, Velonis A, . Evidence for changing intimate partner violence safety planning needs as a result of COVID-19: results from phase I of a rapid intervention. Public Health. 2021;194:11-13. doi:10.1016/j.puhe.2021.02.015 33845272

[3] Pearson I, Butler N, Yelgezekova Z, . Emerging responses implemented to prevent and respond to violence against women and children in WHO European member states during the COVID-19 pandemic: a scoping review of online media reports. BMJ Open. 2021;11(4):e045872. doi:10.1136/bmjopen-2020-045872 33827844 PMC8029039

[4] Thompson N. Reports of domestic, intimate partner violence continue to rise during pandemic. *CityNews*. February 15, 2021. Accessed May 4, 2021. https://www.cbc.ca/news/canada/toronto/domestic-intimate-partner-violence-up-in-pandemic-1.5914344

[5] Dawson M, Sutton D, Zecha A, Boyd C, Johnson A, Mitchell A. #CallItFemicide: understanding sex/gender-related killings of women and girls in Canada, 2020. Canadian Femicide Observatory for Justice and Accountability; 2021. Accessed July 2, 2021. https://femicideincanada.ca/callitfemicide2020.pdf

[6] Swedo E, Idaikkadar N, Leemis R, . Trends in U.S. emergency department visits related to suspected or confirmed child abuse and neglect among children and adolescents aged <18 years before and during the COVID-19 pandemic—United States, January 2019-September 2020. MMWR Morb Mortal Wkly Rep. 2020;69(49):1841-1847. doi:10.15585/mmwr.mm6949a1 33301436 PMC7737689

