# Supplementary material for: Acute Care Visits for Assault and Maltreatment Before vs During the COVID-19 Pandemic in Ontario, Canada
Source: JAMA Health Forum. 2021 Aug 6;2(8):e211983. doi: 10.1001/jamahealthforum.2021.1983 (PMC8796993; doi:10.1001/jamahealthforum.2021.1983)
Supplement: Supplement. — eTable. Diagnosis (ICD-10-CA) Codes Used to Identify Assault and Maltreatment Related ED Visits and Hospitalizations [file jamahealthforum-e211983-s001.pdf]

Supplemental Online Content

Saunders N, Plumptre L, Diong C, et al. Acute care visits for assault and maltreatment before vs during the COVID-19 pandemic in Ontario, Canada. *JAMA Health Forum.* 2021;2(8):e211983. doi:10.1001/jamahealthforum.2021.1983

eTable. Diagnosis (ICD-10-CA) Codes Used to Identify Assault and Maltreatment Related ED Visits and Hospitalizations

This supplemental material has been provided by the authors to give readers additional information about their work.

© 2021 Saunders N et al. *JAMA Health Forum.*

eTable. Diagnosis (ICD-10-CA) Codes Used to Identify Assault and Maltreatment Related ED Visits and Hospitalizations

**Category**	**Code**	**Description**
	R456	Physical violence
	T741	Physical abuse
	X85	Assault by drugs, medicaments and biological substances
	X86	Assault by corrosive substance
	X87	Assault by pesticides
	X88	Assault by gases and vapours
	X880	Assault by carbon monoxide from combustion engine exhaust
	X881	Assault by carbon monoxide from utility gas
	X882	Assault by carbon monoxide from other domestic fuels
	X883	Assault by carbon monoxide from other sources
	X884	Assault by carbon monoxide from unspecified sources
	X888	Assault by other specified gases and vapours
	X889	Assault by unspecified gases and vapours
	X89	Assault by other specified chemicals and noxious substances
	X90	Assault by unspecified chemical or noxious substance
	X91	Assault by hanging, strangulation and suffocation
	X92	Assault by drowning and submersion
	X93	Assault by handgun discharge
	X94	Assault by rifle, shotgun and larger firearm discharge
	X95	Assault by other and unspecified firearm discharge
	X9500	Assault by BB gun discharge
	X9501	Assault by air gun discharge
	X9508	Assault by other specified firearm discharge
	X9509	Assault by unspecified firearm discharge
	X96	Assault by explosive material
	X97	Assault by smoke, fire and flames
	X98	Assault by steam, hot vapours and hot objects
	X99	Assault by sharp object
	Y00	Assault by blunt object
	Y01	Assault by pushing from high place
	Y02	Assault by pushing or placing victim before moving object
	Y03	Assault by crashing of motor vehicle
	Y04	Assault by bodily force
	Y871	Sequelae of assault
	Z0451	Examination and observation following alleged child sexual and physical abuse
Physical assault	Z0458	Examination and observation following other inflicted injury
	Z616	Problems related to alleged physical abuse of child
	T742	Sexual abuse
	Y05	Sexual assault by bodily force
	Z044	Examination and observation following alleged rape and seduction
Sexual assault	Z0450	Examination and observation following alleged adult sexual and physical abuse
	Z614	Problems related to alleged sexual abuse of child by person within primary support group
	Z615	Problems related to alleged sexual abuse of child by person outside primary support group

© 2021 Saunders N et al. *JAMA Health Forum.*

	T73	Effects of other deprivation
	T730	Effects of hunger
	T731	Effects of thirst
	T732	Exhaustion due to exposure
	T733	Exhaustion due to excessive exertion
	T738	Other effects of deprivation
	T739	Effect of deprivation, unspecified
	T740	Neglect or abandonment
	T743	Psychological abuse
	T748	Other maltreatment syndromes
	T749	Maltreatment syndrome, unspecified
Maltreatment	Z629	Problem related to upbringing, unspecified

© 2021 Saunders N et al. *JAMA Health Forum.*
